# Enabling CO_2_ neutral metallurgy for ferrochromium production using bio-based reducing agents

**DOI:** 10.1038/s41598-024-61700-4

**Published:** 2024-05-13

**Authors:** Marcus Sommerfeld, Roberta Botinha, Bernd Friedrich

**Affiliations:** IME Process Metallurgy and Metal Recycling, Institute of RWTH Aachen University, Aachen, Germany

**Keywords:** Chemical engineering, Sustainability

## Abstract

The metallurgical industry is a major source of anthropogenic greenhouse gas emissions. This study explores the replacement of fossil-reducing agents with potentially CO_2_-neutral bio-based reducing agents. Since reducing agents remove oxygen bonded with metal oxides present in the ore, they are a necessity for the production of metallic elements. The investigated metal is chromium, a major part of stainless steel, and therefore a highly relevant element for the transition from a fossil-based energy system to a renewable one. The state-of-the-art smelting reduction and pre-reduction process followed by subsequent smelting using various reducing agents are investigated in this article. The obtained products, metallurgical efficiencies, energy consumption and off-gas generation were compared. While the products produced with bio-based reducing agents are comparable with the reference trials using metallurgical coke regarding the major components in the metal, the concentration of detrimental phosphorus is significantly higher using bio-based reducing agents. The metallurgical efficiency of the process is comparable to the usage of bio-based reducing agents and coke. However, the energy consumption and the generation of off-gas is higher, when coke is replaced by bio-based reducing agents.

Chromium is a relevant alloying element in steel since it improves the corrosion resistivity, hardenability and strength of steel. It is a major constituent of stainless steel which contain between 11 and 25 wt% of chromium^1^. Normally, elements like chromium, manganese, or nickel are not added as pure elements into the steelmaking process, but as ferroalloys containing the alloying element as a major component. Iron and the used reducing agent to separate oxygen from the oxidic raw material are other major elements in ferroalloys^1^. Due to the usage of chromium as an alloying element in stainless steel, the world chromium production per year is constantly rising^2^. Since stainless steel products are used in nearly all end-use sectors^3^ and are relevant for renewable energy systems^4^, it can be expected, that chromium production worldwide will increase even further. The common production process for ferrochromium is carried out in electrically powered furnaces called submerged arc furnaces by a smelting reduction, preceded by an optional pre-reduction process in a solid state^5^. Chromite ore or pre-treated chromite concentrate is used as a chromium carrier and quartz and lime are added as fluxes to improve the properties of the so-called slag, a molten oxide mixture floating on top of the liquid metal^6^. Since chromium and iron are in their oxidic state in chromite the addition of a reducing agent is necessary, normally fossil coal and coke are used^6^, which directly results in the emission of carbon dioxide. It is estimated, that 0.65% of the industrial CO_2_ emissions are due to the usage of fossil-reducing agents in the ferroalloy industry^7^.

Pathways to mitigate fossil carbon emission through the usage of reducing agents in the metallurgical industry include the usage of hydrogen as a reducing agent^8^, renewable bio-based reducing agents^7^, or electrolysis^8^. While electrolysis is only in an experimental phase yet^8^, and hydrogen is nobler than chromium making it an unsuitable reductant^7^, bio-based carbon might be a suitable option, which is CO_2_ neutral, as only the mass of bio-based carbon is used, that is also recultivated^9^. However, there are still knowledge gaps regarding the use of bio-based carbon for the production of ferroalloys, including the supply situation of suitable reductants like charcoal, fluctuating properties of bio-based carbon, the obtainable metal qualities, inferior mechanical properties of charcoal compared to fossil coke and changes in the overall process.

Herein we focus on the usage of four different bio-based charcoals from various sources and a fossil lignite coke as a reference as reducing agents for the production of ferrochromium. The smelting process is investigated and the pre-reduction process followed by a subsequent smelting process. The focus of the study includes the product quality, changes in the energy requirement of the process due to the usage of bio-based reducing agents and changes in the volume of off-gas generated in the process.

## Results and discussion

### Product quality

**Figure 1:**
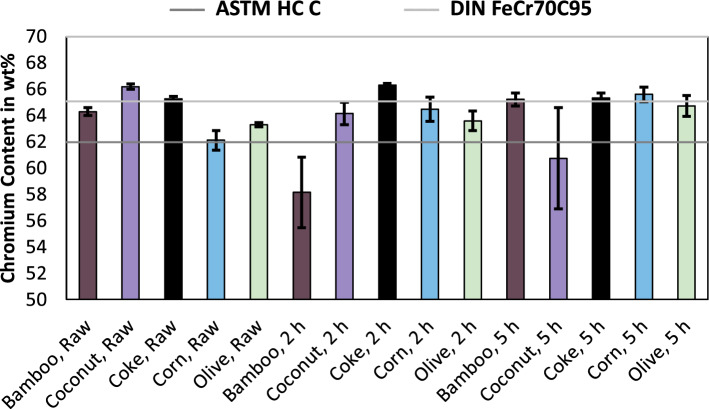
Chromium content in ferrochromium, produced using various reducing agents and for various pre-reduction times. Including the standards for ferrochromium according to ASTM^10^ and DIN^11^.

Figure 1 shows the chromium content in the produced alloy for smelting of raw input materials and smelting of chromite pre-reduced in a solid state for 2 h and 5 h at 1300 °C. Included in the figure is the lower limit for chromium according to the ASTM International (ASTM) standard for high carbon (HC) ferrochromium grade C^10^ and the upper and lower limit according to the “Deutsches Institut für Normung” (DIN) for FeCr70C95 ferrochromium carburé^11^.

**Figure 2:**
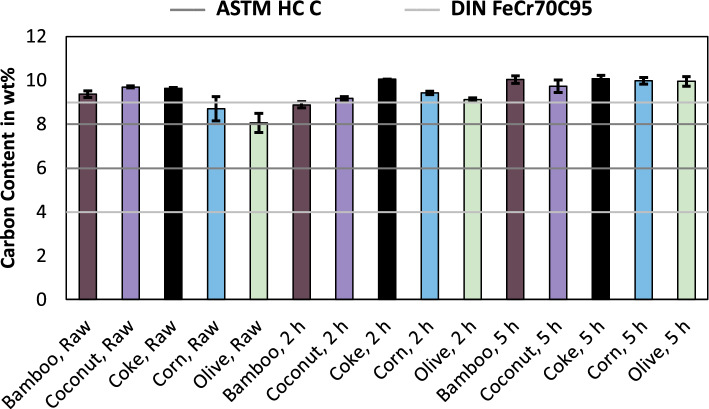
Carbon content in ferrochromium, produced using various reducing agents and for various pre-reduction times. Including the standards for ferrochromium according to ASTM^10^ and DIN^11^.

As can be seen in Fig. 1, the limit according to DIN is higher compared to ASTM. Only the samples produced with raw coconut charcoal, coke and bamboo charcoal and corn charcoal pre-reduced for five hours meet the DIN standard, while the ASTM standard is accomplished by every sample, excluding the trial using corn charcoal, bamboo charcoal used in the pre-reduction process for two hours and coconut charcoal used in the pre-reduction process for five hours. Since at least one trial series per reducing agent satisfied the chromium content according to the ASTM standard, it is assumed, that the usage of bio-based reducing agents does not necessarily result in alloys with an inferior chromium content compared to coke and that deviations in the selected samples or variations in the process like fluctuating raw material compositions resulted in the minor changes. Another possibility can be the increased content of trace elements, which are shown in the following figures. Figure 2 shows the carbon content in the produced alloys and the upper and lower limits according to the previously used ASTM^10^ and DIN^11^ standards.

**Figure 3:**
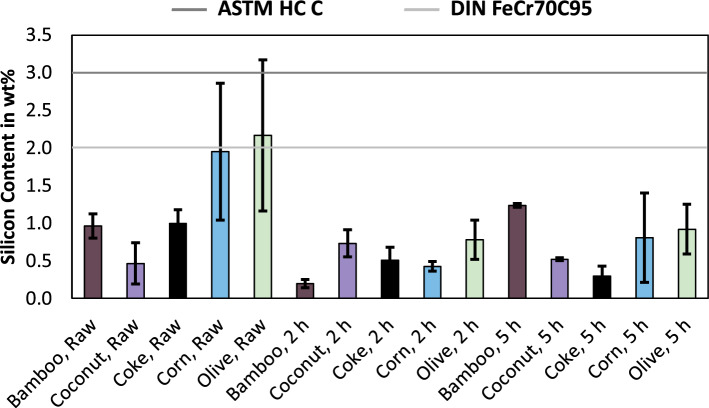
Silicon content in ferrochromium, produced using various reducing agents and for various pre-reduction times. Including the standards for ferrochromium according to ASTM^10^ and DIN^11^.

As shown in Fig. 2, the DIN standard accepts a broader range of carbon in the alloy and has a higher upper limit compared to the ASTM standard. The ASTM standard is not met by any of the trial sets. The DIN standard is only met by the samples produced using corn charcoal and olive charcoal, the other samples exceeded the tolerable carbon content. Since the three trial sets using coke exceeded the standardized carbon content as well, it can be stated, that the high carbon contents are not due to the usage of bio-based carbon and are a general flaw of the used equipment in this study. Since a graphite crucible is used as a smelting vessel, the metal alloy was able to accumulate excess carbon from the crucible in this study, which will be less severe for industrial production. Figure 3 shows the silicon content in the produced alloys, including the upper limits according to ASTM^10^ and DIN^11^.

**Figure 4:**
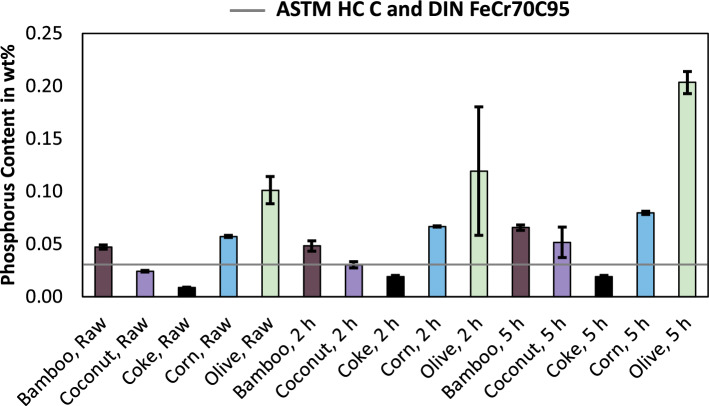
Phosphorus content in ferrochromium, produced using various reducing agents and for various pre-reduction times. Including the standards for ferrochromium according to ASTM^10^ and DIN^11^.

As shown in Fig. 3, the limit for silicon in ferrochrome according to ASTM is one percent higher compared to the DIN standard. Except for the trial set carried out with olive charcoal, all experimental sets are following the DIN standard and therefore also in accordance with the ASTM standard. It is well known, that the reduction of silicon into the alloy is more favourable at higher temperatures^12^, therefore elevated silicon contents in single trials are explainable by temperature deviations within the different trial sets. The composition of the slag also affects the silicon content. Higher levels of basic oxides like CaO or MgO in the slag result in lower silicon contents in the slag, while elevated contents of SiO_2_ result in higher contents of silicon in the metal^13^. Since the addition of oxides in the process remained the same, except for elements that are part of the reducing agents, an influence of the chromite concentrate or fluxes seems neglectable. The analysis of the ash of the reducing agents previously carried out^14^ revealed, that the ash increases in basicity starting with coconut as the most acidic ash, followed by bamboo, corn, olive and coke. Since it was also possible to achieve low silicon contents with the most acidic reducing agent, the composition of the ash of the reducing agents does not have a crucial impact on the silicon content in the alloy. Due to the higher electrical resistivities of charcoal compared to coke^15^, it is necessary to investigate the behavior further on a large scale or with mathematical models, since differences in electrical resistance lead to different temperatures^16^. If the different electrical conductivity of charcoal results in higher temperatures, this could lead to higher silicon contents in the alloy compared to the usage of coke. Figure 4 shows the phosphorus content in the produced alloys and the upper limit according to ASTM and DIN^10,11^, which both tolerate the same amount of phosphorus. Since phosphorus is an impurity in steel^17,18^, a low phosphorus content is desirable in ferroalloys.

While all samples produced with coke meet the requirements for phosphorus in the product, ferrochromium alloys produced using bio-based reducing agents exceeded the tolerable content, except for the trial set carried out with raw coconut charcoal. Although, the set with a pre-reduction time of two hours with coconut charcoal for two hours contains exactly the upper limit. Since bio-based carbon contains higher contents of phosphorus compared to coke^7^, and chromium is less noble than phosphorus^19^, phosphorus will be reduced into the alloy before chromium. Therefore, the high phosphorus contents in the alloys produced with bio-based reducing agents are explainable. As a consequence, the sourcing and selection of bio-based reducing agent regarding the phosphorus content has to be chosen carefully, otherwise, a massive impairment in the product quality is expected. To influence the phosphorus content of bio-based reducing agents, it might be necessary to control the phosphorus uptake of biomass, which is influenced by the use of fertilizers^20^. Another alternative might be the removal of phosphorus from the alloy in a subsequent process, which has been proposed for ferroalloys previously^21–23^, however, this would mean the usage of further fluxes and an additional unit operation.

Another impurity element in ferrochromium is sulfur with a limit of 500 ppm according to the ASTM^10^ and DIN^11^ standard, which is not shown in detail here, since all samples undercut this value significantly with a mean value for coke of 54 ppm and a mean value for the bio-based reducing agents of 46 ppm.

The major component in ferrochromium besides chromium is iron, however, the content of iron is not regulated by the standards used in this work and therefore a detailed description of the iron content is omitted here. The samples produced with bio-based reducing agents had an iron content between 21.73 and 32.71 wt% while the samples produced with coke had an iron content between 21.80 and 24.85 wt%.

### Metallurgical efficiency

**Figure 5:**
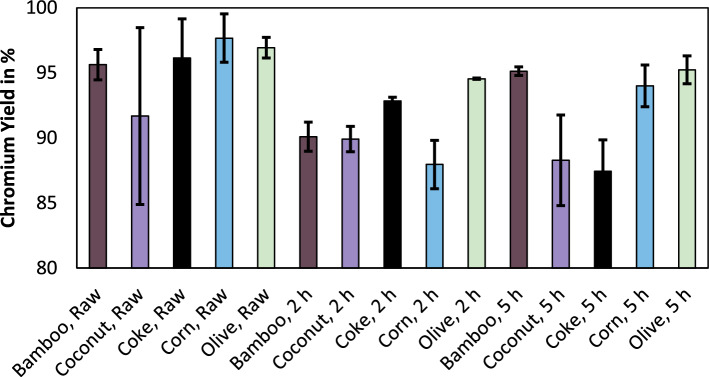
Chromium yield using various reducing agents and for various pre-reduction times.

The metallurgical efficiency of metallurgical processes can be described as the distribution of the target element into a targeted phase. In the case of ferrochromium production, this would be the distribution of chromium into the metal phase. Figure 5 shows the chromium yield obtained in this study.

The mean yield of the trials was between 87.4 and 97.7%. Based on experience, the yield can deviate significantly for laboratory trials in a submerged arc furnace. Therefore, the dispersed results are explainable. However, there seems to be no evidence that bio-based reducing agents or coke are superior when it comes to the yield of chromium. The iron yield is omitted here. Since the yield for the less noble chromium is relatively high, it is clear on a thermochemical basis that the yield for iron is also high. The mean iron yield using bio-based reducing agents was 98.9%, while the mean yield when coke was used as a reducing agent was 98.2%.

### Specific energy demand

**Figure 6:**
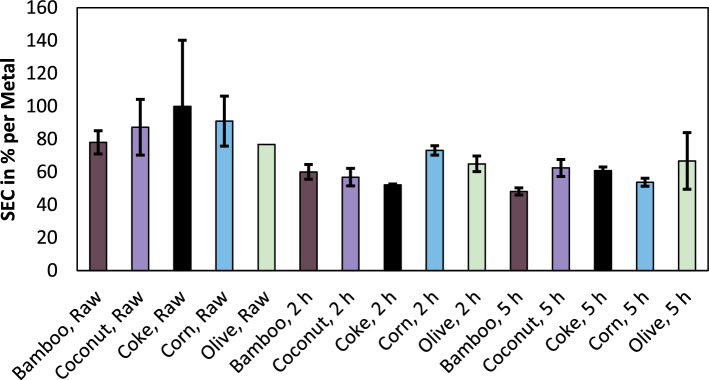
Relative specific electric energy consumption of smelting trials for various reducing agents and various pre-reduction times.

To the economic viability of the process, the energy demand to produce the desired product is of high relevance. In a submerged arc furnace, the consumption of electricity in relation to the mass of produced product, is the specific electric energy consumption (SEC). Figure 6 shows the relative SEC for the smelting of raw feed material and pre-reduced feed material. The feed material was not charged into the furnace directly after the pre-reduction, but after cooling to room temperature. Instead of the absolute values, the SEC is normalized compared to the specific energy demand used for the trials with coke and without pre-reduction. The trials with coke consumed 29,436 kWh/t_Alloy_. Since the trials were carried out in a laboratory-scale furnace with a low energy efficiency, those values are not comparable with industrial values, which are normally in a range between 3000 and 3500 kWh/t_Alloy_^24^.

One well-known fact is, that the SEC decreases if the chromite is pre-reduced for the smelting process^25^, which is also visible in the presented results. Otherwise, it has to be noted, that the energy consumption in such small-scale experiments is highly variating, clearly visible for the trial with coke and without pre-reduction, which has the highest SEC in one trial and the lowest SEC as well in another trial compared to the other reducing agents without pre-reduction. Neglecting the coke trial with the highest energy consumption, coke results in the lowest energy consumption for raw feed material and material pre-reduced for two hours. For trials pre-reduced for five hours, coke only delivered average results, since the usage of bamboo- and corn charcoal resulted in lower specific energy demand. Therefore, especially for processes without or only a short pre-reduction, the specific electric energy consumption increases, if bio-based reducing agents are used, while at longer pre-reducing times the SEC is more balanced. One explanation could be, that the bio-based reducing agents result in a better pre-reduction, which was observed previously using the same material^14^.

Since the measuring system malfunctioned during one trial with olive, the SEC is only shown for one trial and without error bars in that case. As another inaccuracy, it has to be noted, that the silicon content is not constant in this work as shown in Fig. 3, due to reasons not related to the properties of reducing agents as described above. In general, the energy consumption in the smelting process is higher if more silicon is reduced during the process^12^.

**Figure 7:**
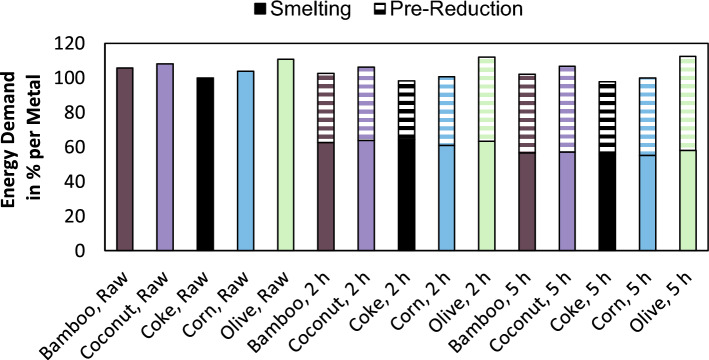
Relative specific energy consumption of smelting and pre-reduction for various reducing agents and various pre-reducing times according to a simulation with a constant addition of fixed carbon.

To also include the energy necessary for the pre-reduction process, thermochemical modeling of both processes was carried out. The detailed procedure is described in the supplementary material. Figure 7 shows the normalized specific energy demand. The usage of coke without pre-reduction is again defined as the base scenario with a specific energy demand of 4217 kWh/t_Alloy_. This value is still higher than the previously presented energy demand. However, since the trials were carried out in a direct current electric arc furnace, the simulation also considers a direct current electric arc furnace, which does not use the latent heat contained in the off-gas to pre-heat the charged raw materials. The specific electric energy consumption for direct current arc furnaces is around 4200 kWh/t_Alloy_^6^.

Comparing the simulation with raw feed material, coke has the lowest energy demand, followed by corn-, bamboo-, coconut- and olive charcoal. The same order is observable if the reducing agents are listed by a descending fixed carbon content or an ascending content of volatile matter and moisture. Sorting the reducing agents only by the volatile matter content does not result in the same trend, since bamboo charcoal has a lower volatile matter content than corn, but a significantly higher moisture content. Due to the pre-reduction, the SEC is reduced to 55.2–64.4%, while the total energy demand is reduced by 1.5–3.7% with a mean value of 2.6%, neglecting olive charcoal, where the pre-reduction and smelting process consumes 1.1% and 1.7% more energy compared to the direct smelting. Therefore, from a total energy point of view, the two-stage process does only save a small fraction of energy. From a greenhouse gas emission perspective, it is highly relevant how the energy is supplied in the process. A submerged arc furnace consumes electrical energy, which can potentially be renewable. Rotary kilns are commonly used for pre-reduction, which are heated by gas-, oil- or coal burners^7^, which can be also renewable in the case of hydrogen or bio-based materials, but are typically powered with fossil fuels. Therefore, the footprint of the energy sources needs to be considered to determine if the pre-reduction process is aiding the production of CO_2_ neutral or minimized ferroalloys.

### Off-gas generation of the process

**Figure 8:**
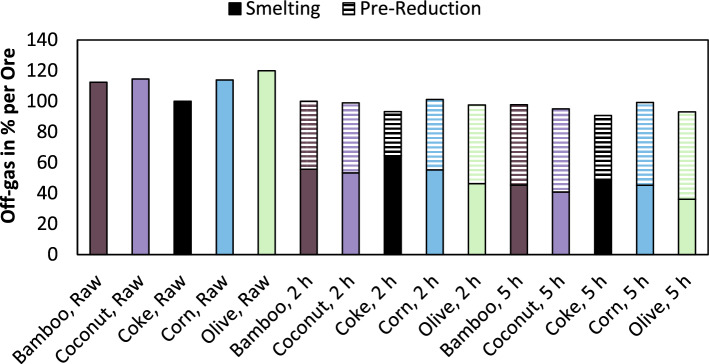
Relative off-gas generation of smelting and pre-reduction for various reducing agents and various pre-reduction times.

One relevant aspect for existing metallurgical plants considering the usage of bio-based reducing agents is the amount of off-gas generated in the process, especially for manufacturers, operating close to the design capacity of their off-gas treatment infrastructure. Figure 8 shows the normalized off-gas volume per treated chromite concentrate mass according to the thermochemical model. The base scenario is the usage of coke without pre-reduction resulting in an off-gas volume of 2831 m^3^/t_Ore_. It has to be emphasized, that the off-gas volume is given in m^3^ and not in Nm^3^. However, this volume of off-gas is only based on the gases generated due to reactions or devolatilization of feed material.

The usage of bio-based reducing agents results in an increase of off-gas by 12–14% for bamboo-, coconut- and corn charcoal. Although, it is even 20% for the usage of olive charcoal, which is also the material with the highest volatile matter and moisture content. Due to the pre-reduction, the total emission of off-gas is reduced. For coke, the off-gas emissions are reduced by 6.7 and 9.2%. In the case of bio-based reducing agents, the highest reductions are observable for olive charcoal, with 22.3 and 26.7%. For coconut charcoal, the reduction is 6.7 and 19.4% while for the other reducing agents, the reduction is 12.4–15.5%.

## Conclusion

Bio-based carbon is a viable option to lower the CO_2_ footprint to produce ferroalloys. Besides deficiencies regarding the mechanical properties and fluctuating properties of bio-based reducing agents, which were not addressed in this paper, there are more challenges to overcome. However, this study showed, that there are also performance indicators not negatively influenced by the usage of bio-based reducing agents like the:

- Chromium yield
- Iron yield
- Bulk chemical composition considering chromium, iron, carbon and silicon
- Specific electric energy consumption for smelting of pre-reduced chromite
- Sulfur content in the alloy

Significant disadvantages resulting from the usage of bio-based carbon addressed in this study include the following points:

- Phosphorus content in ferrochromium is significantly higher using bio-based reducing agents
- Total energy consumption of the smelting process and the pre-reduction smelting process is higher using bio-based reducing agents (up to 12%)
- Higher off-gas volume due to the usage of bio-based reducing agents (up to 20%), even though this fact is mitigated slightly, if a pre-reduction process is carried out

Since the energy consumption and off-gas volumes are closely related to the volatile matter and moisture content, further heat treatment of bio-based carbon might be an option to overcome those issues, however, this only transfers the energy consumption and off-gas generation from the ferrochrome producer to the charcoal producer. Considering the energy demand of smelting using raw feed material and the total energy demand of pre-reduction and subsequent smelting, the energy demand is increasing according to the thermochemical model for decreasing fixed carbon contents. The lowest energy demand is predicted for the usage of coke, followed by corn, bamboo, coconut and olive charcoal. Small deviations to this generalization are observable, if only the specific electric energy consumption for smelting pre-reduced material is investigated. After a pre-reduction time of two hours, the relative electric energy consumption is 61.1% in the lowest case using corn charcoal and 64.4% in the highest case using coke. After a pre-reduction time of five hours, the lowest electric energy demand is observable using corn charcoal as well with 55.2%, while the highest electric energy demand is predicted for the usage of olive charcoal with 58.1%.

A more significant problem is the high phosphorus content in ferrochromium. Independent of the usage of raw materials or pre-reduced material, metal produced using coke has the lowest phosphorous content, followed by coconut, bamboo, corn and olive charcoal. Even though an approach to reduce the phosphorus uptake in the alloy during the smelting reduction process is not an option due to thermochemical reasons, other approaches have to be investigated more thoroughly, which are also accompanied by disadvantages.

- Selection of bio-based reducing agents with a low phosphorus content (difficult due to the high demand for bio-based reducing agents)
- Refinement of the alloy after the smelting reduction process (increases the production time, consumes further raw materials and energy and requires new infrastructure)
- Development of steel grades, that tolerate higher phosphorus contents (difficult, due to various negative impacts of phosphorus on the properties of steel)

## Materials and methods

Pre-reduction trials were carried out in clay crucibles made by Atlantic Schmelztiegel GmbH, placed in a Nabertherm HT 16/18 furnace made by Nabertherm GmbH under an argon atmosphere. Pre-reduction was carried out at 1300 °C for two and five hours. Smelting trials were carried out at 1700 °C ± 50 °C in a direct-current laboratory-scale electric arc furnace using graphite crucibles with a volume of two Liter, the furnace details were described previously^26,27^. Chromite concentrate from Turkey was used as a chromium carrier. The concentrate was thoroughly described in a previous publication^28^. The reducing agents used in this study were bamboo charcoal, coconut shell charcoal, lignite coke, corn cob charcoal and olive pomace charcoal. The proximate-, ultimate- and ash analysis is included in the supplementary material. The particle size analysis were previously shown^14^. Photographs of the used raw material are shown in the supplementary material.

Before smelting the chromite and reducing agent, 1000 g of oxides (8.32 wt% Al_2_O_3_, 35.02 wt% CaO, 16.63 wt% MgO and 40.03 wt% SiO_2_) were molten into a synthetic slag to achieve a homogenous temperature in the furnace. The slag composition is based on the amount of Al_2_O_3_, CaO, MgO and SiO_2_ introduced via the concentrate and fluxes and is normalized to 100 wt%. Afterward, 2000 g of as received chromite concentrate was charged into the synthetic slag with manually crushed reducing agents and fluxes. Based on the chromite concentrate mass, 18 wt% of fixed carbon and 35 wt% of lime and 35% of quartz as fluxes were charged into the furnace. Oxidic raw materials were Al_2_O_3_ (99.5 wt% Al_2_O_3_) from Nabaltec AG, burnt lime (94.6 wt% CaO) from Rheinkalk GmbH, MgO (> 96 wt% MgO) from Thermo Fisher Scientific Inc. and quartz (> 98 wt% SiO_2_) from Quarzwerke GmbH. For calculations and simulations, all oxides were considered to be 100% pure. Pre-reduction and subsequent smelting of pre-reduced material were carried out using the same mass of chromite concentrate and reducing agent, while the fluxes were only added in the smelting step. Every trial was carried out two times. The regular power input during the smelting trial was around 10 kW, depending on the assessment of the operator. The mean smelting time of raw feed material was two hours and 8 min, while the mean smelting time of pre-reduced material was one hour and 36 min. In the supplementary material, it is described how the additions of reducing agents and fluxes were determined. Slag samples were analyzed using wavelength dispersive x-ray fluorescence spectroscopy (Axios^max^) made by Malvern Panalytical. Carbon and sulfur in the alloy were analyzed using an ELTRA CS2000 system based on a total combustion method, made by ELTRA GmbH. The iron-, silicon- and phosphorus content in ferrochromium was analyzed by a certified laboratory following DIN EN ISO 11885^29^, the chromium content was analyzed following ISO 4140^30^. FactSage™ 8.2^31^ was used for thermochemical simulation. The yield was calculated using Eq. (1), since in the laboratory-scale batch process, losses occur due to splashing of liquid phases and dusting of raw materials, which impair the results significantly if the yield is calculated on an input and output base. In Eq. (1), η_x_ is the yield for element x in % and ${\text{m}}_{{\text{x}}_{\text{y}}}$ is the mass of element x in the phase y.

1 $${\upeta }_{{\text{x}}} {\text{ = 100\% }} \cdot \frac{{{\text{m}}_{{{\text{x}}_{{{\text{Metal}}}} }} }}{{{\text{m}}_{{{\text{x}}_{{{\text{Metal}}}} }} {\text{ + m}}_{{{\text{x}}_{{{\text{Slag}}}} }} }}$$

## Additional information

Supplementary information accompanies this paper at 10.1038/s41598-024-61700-4, including photographs of the used raw materials, descriptions of the method of the thermochemical simulation, the slag design, the reducing agent analysis and the determination of the mass of required reducing agents, partially based on the following publications^7,14,32–39^.

### Supplementary Information

Supplementary Information.

## Data Availability

The datasets generated during and/or analyzed during the current study are available from the corresponding author on reasonable request.
