# Supplementary material for: Enabling CO2 neutral metallurgy for ferrochromium production using bio-based reducing agents
Source: Sci Rep. 2024 May 13;14:10878. doi: 10.1038/s41598-024-61700-4 (PMC11091206; doi:10.1038/s41598-024-61700-4)
Supplement: Supplementary file 1 — Supplementary Information. [file 41598_2024_61700_MOESM1_ESM.pdf]

Supplementary Material

Enabling CO_2 Neutral Metallurgy for Ferrochromium Production Using Bio-Based Reducing Agents

Marcus Sommerfeld[1], Roberta Botinha[1], Bernd Friedrich[1]

IME Process Metallurgy and Metal Recycling, Institute of RWTH Aachen University, Aachen, Germany. Correspondence and requests for materials should be addressed to M.S. (email: msommerfeld@ime-aachen.de)

Photographs of the as Received Raw Materials

Photographs of the as received raw materials are presented in Supplementary Figure S1.

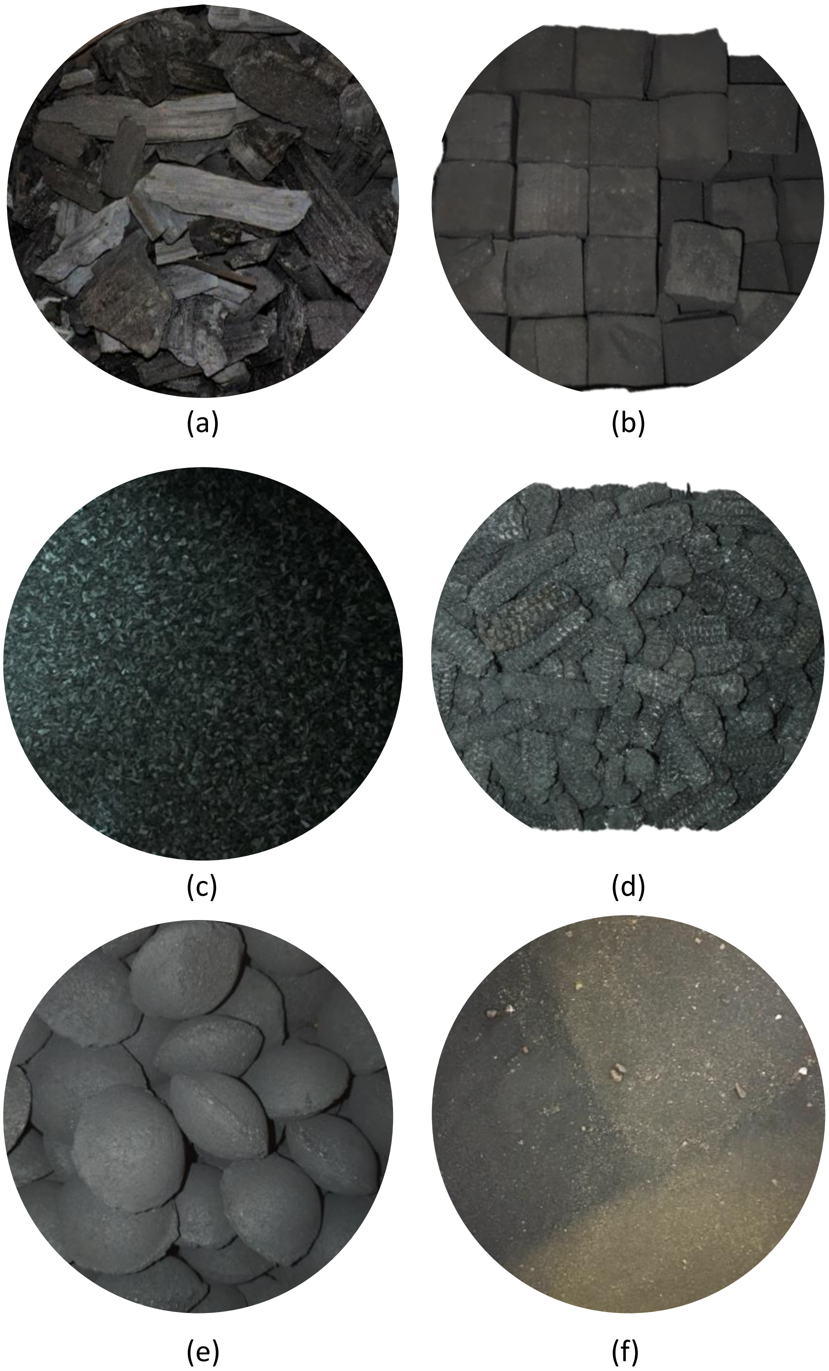

Supplementary Figure S1. Photographs of bamboo charcoal (a), coconut charcoal (b), lignite coke (c), corn charcoal (d), olive charcoal (e) and chromite concentrate (f).

Mineralogical Reconciliation of the Used Chromite

Based on an approach suggested by Sweeten et al.[1], the mineralogical composition of the chromite concentrate is included in the thermochemical simulation. Chemical concentration, mineralogical assessment and iron specification of the chromite concentrate have been previously described[2]. Since the chromite concentrate used in this study has a higher MgO to Al_2O_3 ratio, the approach was adapted. The following steps have been carried out, to obtain a mineralogical composition suitable for the thermochemical model:

1. Moisture present in the concentrate is taken from thermogravimetry results up to a temperature of 250 °C. For temperatures above 250 °C it is expected, that brucite or goethite decompose as well[3,4], while lizardite decomposes at 635 °C[5].

2. Based on the suggestion of Sweeten et al.[1], the difference between the measured concentration of 97.92 wt% was normalized to 100 wt% by increasing the amount of MgO, Al_2O_3, SiO_2 and CaO proportionally. Without normalizing the composition, the energy consumption based on the simulation would underestimate the actual energy consumption, while increasing the share of chromium and iron would overestimate the alloy production.

3. Carbon in the chromite concentrate is attributed to dolomite ($CaMgC_2O_6$) and aragonite ($CaCO_3$) according to the ratio determined by quantitative evaluation of minerals by scanning electron microscopy (QEMSCAN®). Aragonite was selected, since it is thermochemically stable at room conditions, even though calcite and aragonite were both identified via Raman microscopy.

4. Residual calcium is assigned to olivine. Identified diopside by Raman microscopy is neglected because attributing calcium to diopside would result in an underestimated olivine concentration compared to the QEMSCAN® measurement.

5. Sodium and potassium are equilibrated with aluminum, silicon and oxygen as a feldspar solid solution ($NaAlSi_3O_8$-$KAlSi_3O_8$).

6. The brucite ($Mg(OH)_2$) concentration is included as identified by QEMSCAN®.

7. The Fe-oxides based on QEMSCAN® are included as goethite ($Fe_2O_3(H_2O)$).

8. The MgAl-Silicate value based on QEMSCAN® is introduced as chlinochlorite ($Mg_5Al_2Si_3O_{10}(OH)_8$). Since dozyite ($Mg_7Al_2(Al_2Si_4)O_{15}(OH)_{12}$) was identified by Raman microscopy, it would have been logical to select dozyite instead, however, data for dozyite is not included in the FactSage™ database.

9. Residual hydrogen is assigned to lizardite ($Mg_3Si_2O_5(OH)_4$), which was identified by Raman microscopy. Starting the assigning of hydrogen with lizardite was not possible, since this would have resulted in a too high silicon content compared to the chemical analysis.

10. Residual silicon is attributed with residual calcium and magnesium into an olivine solid solution (Ca_2SiO_4-Mg_2SiO_4).

11. Titanium, vanadium, sulfur and phosphorus are all assigned to the thermochemically stable phases according to FactSage™. Titanium is assigned to an ulvospinel ($(FeO)_2TiO_2$), vanadium is assigned to an FeV_2O_4 phase, sulfur is assigned to an FeS phase and phosphorus is assigned to an $Mg_3P_2O_8$ phase.

12. Residual aluminum, chromium, iron, magnesium, manganese and oxygen is equilibrated using FactSage™ resulting in the main spinel phase.

Reconciliation of Reducing Agents

To include the different reducing agents as accurately as possible in the thermochemical simulation, a reconciliation approach is applied, based on the ultimate-, proximate-, ash-, chlorine and x-ray diffraction (XRD) analysis. The proximate-, ultimate- and chlorine analysis are shown in Supplementary Table S1.

Supplementary Table S1. Proximate-, ultimate- and chlorine analysis (after[6]).

In wt%	Proximate Analysis				Ultimate Analysis					Cl
**Reducing Agent**	**Fixed Carbon**	**Total Moisture**	**Ash**	**Volatile Matter**	**C**	**H**	**N**	**S**	**O**	
**Bamboo Charcoal**	80.4	11.6	3.9	4.1	79.73	1.21	0.40	0.08	3.12	0.12
**Coconut Charcoal**	72.1	8.7	7.2	12.1	73.84	1.24	0.39	0.06	8.60	0.07
**Coke**	87.5	0.5	9.0	3.0	89.0	0.4	0.4	0.5	0.7	0.03
**Corn Charcoal**	81.3	4.6	4.8	9.3	81.62	2.30	0.63	0.04	5.99	0.51
**Olive Charcoal**	63.7	7.2	12.6	16.4	67.54	1.60	1.16	0.05	9.79	0.43

The ash analysis of the reducing agents is shown in Supplementary Table S2.

Supplementary Table S2. Ash analysis (after[6]).

Reducing Agent	Ash Composition in wt%							
	**Al_2O_3**	**CaO**	**Fe_2O_3**	**K_2O**	**MgO**	**Na_2O**	**P_2O_5**	**SiO_2**
**Bamboo Charcoal**	2.8	2.8	3.1	35.6	3.9	<0.01	6.3	30.6
**Coconut Charcoal**	4.1	23.0	4.1	7.7	3.6	2.9	1.6	46.8
**Coke**	3.1	35.4	10.9	0.8	15.8	6.7	0.2	2.2
**Corn Charcoal**	0.6	1.8	1.5	46.1	3.4	<0.01	6.4	15.8
**Olive Charcoal**	2.2	16.8	2.0	34.2	3.4	1.6	7.1	8.0

The analysis has been presented in a previous publication[6], except the XRD analysis. Supplementary Figure S2 shows the XRD data of carbonaceous reducing agent ash and the three main phases per sample. Supplementary Table S3 also lists the name of the phases and the used powder diffraction file (PDF) shown in Supplementary Figure S2.

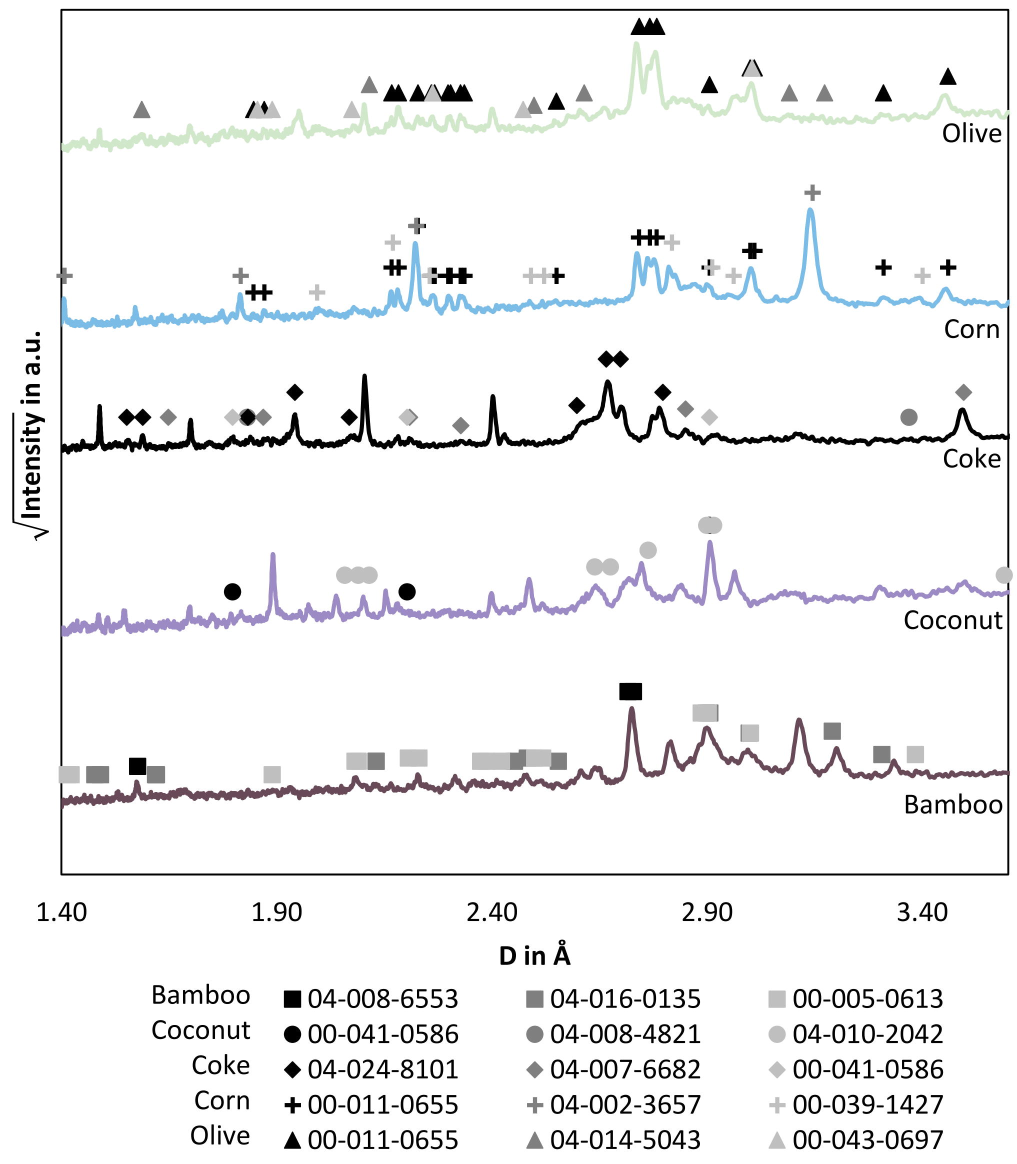

Supplementary Figure S2. X-ray diffraction analysis of reducing agent ash.

Supplementary Table S3. Major phases present in the reducing agent ash.

Reducing Agent	PDF-Number	Phase
**Bamboo**	04-008-6553	α-$KAlO_2$
	04-016-0135	Clinoenstatite
	00-005-0613	Arcanite
**Coconut**	00-041-0586	Ankerite
	04-008-4821	Quartz
	04-010-2042	Calciumphosphate
**Coke**	04-024-8101	Brownmillerite
	04-007-6682	Anhydrite
	00-041-0586	Ankerite
**Corn**	00-011-0655	Potassium Carbonate Sesquihydrate
	04-002-3657	Sylvite
	00-039-1427	Potassium Calcium Silicate
**Olive**	00-011-0655	Potassium Carbonate Sesquihydrate
	04-014-5043	Megakalsilite
	00-043-0697	Calcite (Mg-bearing)

Based on this information, the following stepwise approach was used for the reconciliation.

1. The total moisture content of the proximate analysis is included as $H_2O_{(l)}$.

2. The fixed carbon content of the proximate analysis is included as $carbon_{(graphite)}$.

3. The ash composition is multiplied by the ash content to obtain the amount of each element in the raw coal. Instead of the oxides as presented, sulfur is included as K_2SO_4 for the bio coals, while $CaSO_4$ is included for coke. Chlorine is included as KCl. For samples containing potassium carbonate sesquihydrate ($K_2CO_3(H_2O)_{1.5}$) according to XRD as a main phase (corn and olive), the chlorine content is subtracted from the loss on ignition (LOI), the residual LOI is assumed to occur, due to the decomposition of potassium carbonate sesquihydrate to K_2O. Residual potassium is included as K_2O.

4. Due to the presence of transition metals (iron), the program is not able to simulate an equilibrium for every ash. To allow the balancing of Fe^{2+} and Fe^{3+}, an oxygen activity of 10^{-10} is set as a boundary condition. This has no significant impact on the results since less than approximately 10^{-22} g of oxygen is deducted from the system per 100 g of reductant, due to this approach. The ash is then equilibrated using FactSage™.

5. The total carbon content determined by the ultimate analysis is subtracted by the fixed carbon content of the proximate analysis, and the hydrogen-, nitrogen- and oxygen content are equilibrated using FactSage™ as a gas mixture. For minerals, where the presence of potassium carbonate sesquihydrate was observed, the carbon-, hydrogen- and oxygen amount released during the decomposition of $K_2CO_3(H_2O)_{1.5}$ to K_2O is deducted as well. In addition, the oxygen bound as SO_3 in $CaSO_4$ and K_2SO_4 is deducted, since these sulfates do not decompose during the ashing of coal, but will decompose at the temperature used for the ultimate analysis[7].

This approach is only an approximation since the inorganic components in biomass are at least partially occurring as free radicals[8], while discrete particles like KCl start to form during the decomposition of organic matter[9]. Since charcoal will not be completely decomposed compared to ash, it will not be possible to reconcile a completely accurate composition including the

deviation between discrete compounds and radicals present in the raw material. Also, the approach describing the volatile matter simplifies the composition drastically, since the volatiles might be adsorbed as liquid compounds[10] and solid carbohydrates might be present in the charcoal as well. For both cases, differentiation is very difficult and the dataset for those compounds is incomplete in FactSage™. It would also be possible to equilibrate the volatile matter as liquids or as liquid and gas, but this is not possible for all reducing agents. Therefore, it was decided to equilibrate all reducing agents as gas.

Simulation of the Smelting Process

The smelting process was simulated with the input streams and parameters used in the trials. The reconciled compositions of the reducing agents and chromite concentrate as described above were introduced into the system. Besides the addition of reducing agents equal to a fixed carbon addition of 18 wt% in relation to the chromite concentrate mass, a variable amount of graphite was added as well. Since the trials were carried out using a graphite crucible, a non-measurable amount of graphite reacted with the melt. The simulation showed that an addition of 4 wt% graphite results in a similar metal composition as achieved in the trials, therefore, 4 wt% of graphite was added in the thermochemical model.

Simulation of the Pre-Reduction and Smelting Process

The simulation of the pre-reduction process was simulated similarly compared to the smelting process. Chromite concentrate and carbonaceous reducing agents were used in the pre-reduction process. The pre-reduced material was introduced into the smelting stage, with the same temperature as the pre-reduction temperature of 1300 °C, therefore, heat losses due to the transfer to the furnace were neglected. Graphite and fluxes were added in the smelting stage. To include the experimental results of the pre-reduction, which in reality does not reach thermal equilibrium, compared to the thermochemical simulation, the amount of reacted reducing agent had to be adjusted. Therefore, the pre-reduction was simulated with variable reducing agent additions. It was assumed, that the mass after pre-reduction of the trials, is equal to the mass of the simulation including a reacted fraction of the reducing agent with the concentrate and an unreacted fraction as shown in equation (1). The mass loss of the reducing agent (Δm_{Coal}) was determined experimentally while the used reducing agent mass for a constant fixed carbon addition of 18 wt% ($m_{Coal,\ CFix=18\ wt\%}$) minus the reacted reducing agent mass ($m_{Coal,\ reacted}$) is the amount of reducing agent, that did not react with the chromite concentrate and only devolatilized at 1300 °C. The simulation was iterated for several amounts of reacted reducing agents, until equation (1) resulted in the same value as the results from the trial.

$$m_{Total} = m_{Simulation,\ for\ m_{Coal,\ reacted}} + \Delta m_{Coal} \cdot (m_{Coal,\ C_{Fix}=18\ wt\%} - m_{Coal,\ reacted}) \qquad (1)$$

Based on those assumptions, Supplementary Figure S3 shows the amount of reacted reducing agent, used for the simulation of the pre-reduction.

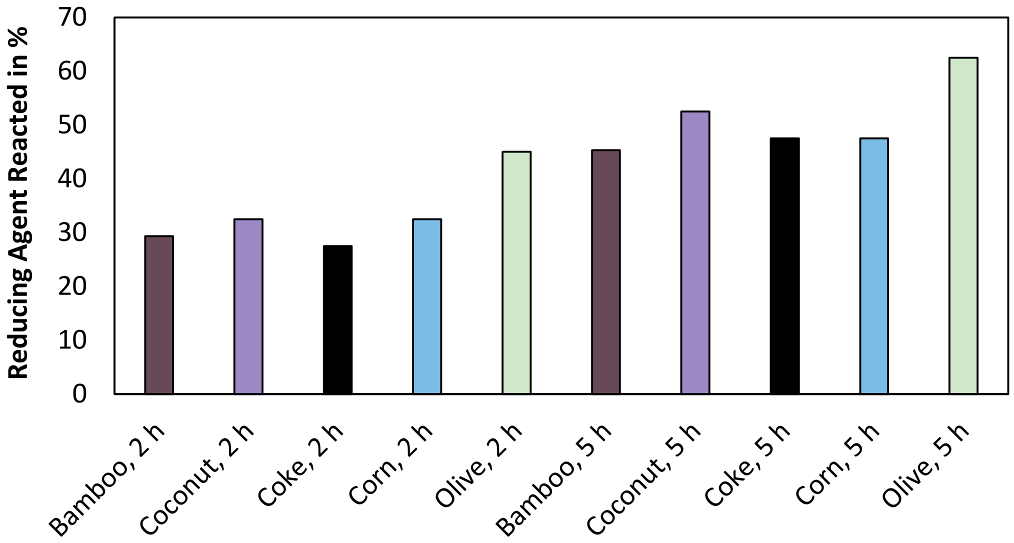

Supplementary Figure S3. Assumed percentage of reacted reducing agent for various pre-reduction times used in the thermochemical simulation.

Determination of the Necessary Amount of Fluxes

To determine the mass of fluxes necessary, a thermochemical simulation with FactSage™ was carried out. As fluxes, lime and silica were considered. To simplify the calculation, minor elements in the concentrate were neglected. In addition, chromium oxide and iron oxides were neglected as well, since they are reduced during the process into the metal phase. Therefore, only Al_2O_3, CaO, MgO and SiO_2 were included from the chromite concentrate as shown before[2]. Supplementary Figure S4 shows the liquidus temperature of the simplified and reduced slag, for various lime and silica additions.

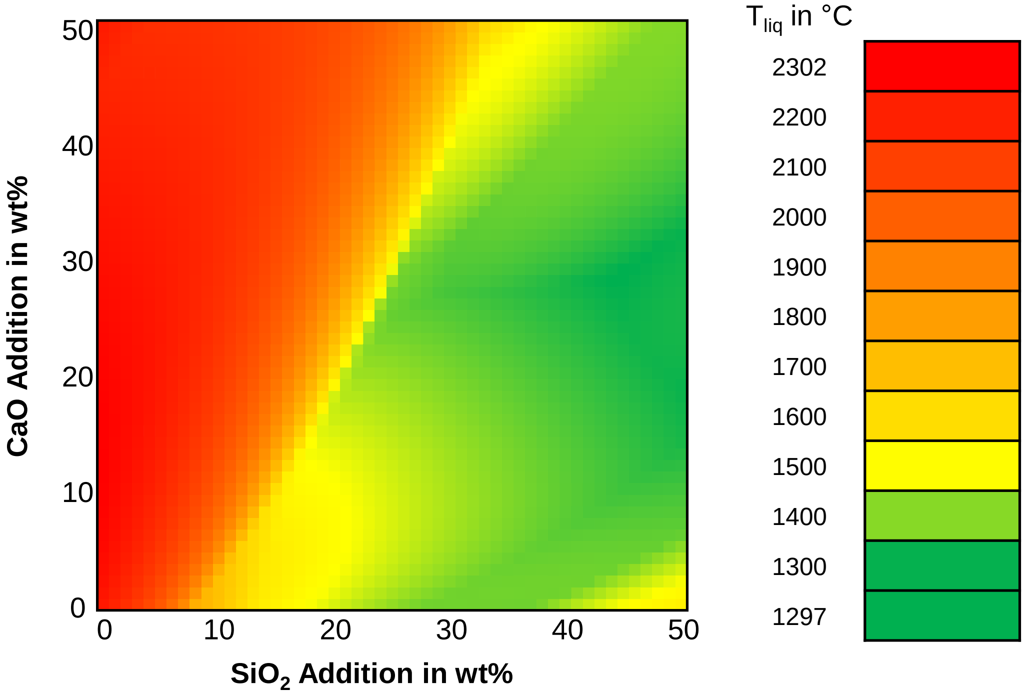

Supplementary Figure S4. Liquidus temperature of reduced and simplified slag for various lime and silica additions.

Without the addition of fluxes, the liquidus temperature and consequently the process temperature exceeds 2200 °C. The addition of silica is necessary to decrease the liquidus temperature of the slag, while at high silica additions, it is also possible to add lime, without

increasing the liquidus temperature severely. In theory, the addition of over 20 wt% silica results in a slag, with a liquidus temperature of 1400 °C, however, residual chromium contents seemed to increase the liquidus temperature during pre-trials. Therefore, higher temperatures were used. Pre-trials showed, that a process temperature of 1700 °C results in a suitable slag with a good metal and slag separation, which was used in the main trials as well.

Another important property influenced by the addition of fluxes is the silicon content in ferrochromium. A simulation was carried out to determine the silicon content in the alloy after the smelting process. Therefore, iron oxides and chromium oxides were also included, compared to the simulation shown in Supplementary Figure S4. Since in the trials, the metal and slag are in contact with a graphite crucible and graphite electrode, the simulation was carried out in equilibrium with graphite. As a temperature, 1700 °C was selected as well. The silicon content in ferrochromium for varying lime and silica additions is shown in Supplementary Figure S5.

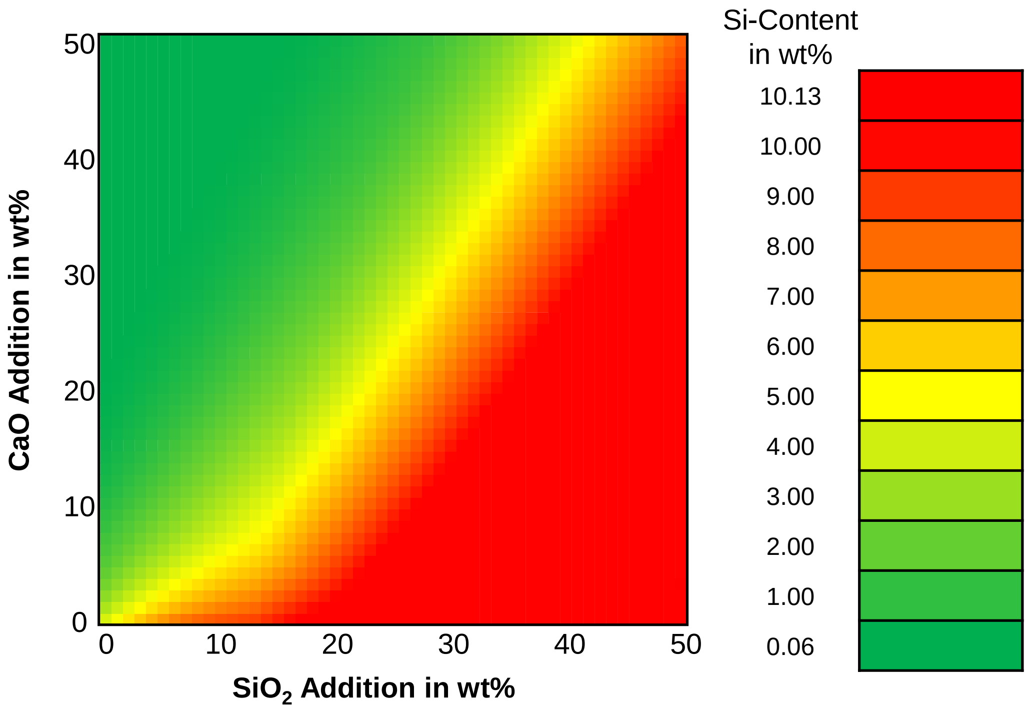

Supplementary Figure S5. Silicon content in ferrochromium in equilibrium with graphite at 1700 °C for varying SiO_2 and CaO additions.

As discussed in the article, the silicon content should be below 2 wt% or 3 wt%, depending on the applied standard. The addition of silica will yield high silicon contents in the metal, while the addition of lime decreases the silicon content. However, the sole addition of lime will result in a slag, with a liquidus temperature above the envisaged process temperature as shown in Supplementary Figure S4. Therefore, it is necessary to add both silica and lime. Comparing Supplementary Figure S5 and Supplementary Figure S4, a slightly higher addition of silica compared to lime, seems to result in a liquid slag and a reasonable silicon content in the alloy. However, pre-trials carried out with a silica addition of 35 wt%, a lime addition of 20 wt% and the addition of coconut shell charcoal resulted in an alloy containing 11.63 wt% silicon exceeding the limits. Further pre-trials with silica additions of 35 wt% and lime additions of 35 wt% resulted in reasonable silicon contents and a suitable slag regarding the process temperature and metal and slag separation. Therefore, the experiments in the article were carried out with those flux additions.

Determination of the Necessary Amount of Reducing Agents

Based on the amount of chromium and iron oxides in the chromite concentrate, it is possible to calculate the stoichiometric amount of carbon necessary for the reduction of those oxides. In

theory, 14.08 wt% of carbon is necessary compared to the mass of the chromite concentrate. Since ferrochromium dissolves carbon, it is estimated that an addition of 3.52 wt% of carbon is necessary to produce an alloy containing 8 wt% of carbon. Therefore a total carbon addition of 17.60 wt% is necessary. Pre-trials were carried out with higher and lower carbon additions. Lower carbon additions resulted in rapid consumption of the graphite crucible. Higher carbon additions resulted in unreacted reducing agents floating on the slag. After pre-trials, 18 wt% of a fixed carbon addition was selected as a suitable carbon addition.

References

1. Sweeten, N. J., Verryn, S., Oberholzer, J. & Zietsman, J. H. Chrome Ore Mineralogy and the Furnace Mass and Energy Balance. *J S Afr Inst Min Metall* **118**; 10.17159/2411-9717/2018/v118n6a12 (2018).
2. Sommerfeld, M. & Friedrich, B. Proposition of a Thermogravimetric Method to Measure the Ferrous Iron Content in Metallurgical-Grade Chromite. *Minerals* **12,** 109; 10.3390/min12020109 (2022).
3. Gordon, R. S. & Kingery, W. D. Thermal Decomposition of Brucite: II, Kinetics of Decomposition in Vacuum. *J American Ceramic Society* **50,** 8–14; 10.1111/j.1151-2916.1967.tb14962.x (1967).
4. Ammasi, A. Effect of Heating Rate on Decomposition Temperature of Goethite Ore. *Trans Indian Inst Met* **73,** 93–98; 10.1007/s12666-019-01806-w (2020).
5. Weber, J. N. & Greer, R. T. Dehydration of Serpentine: Heat of Reaction and Reaction Kinetics at p_{H2O}=1 atm. *Am Mineral* **50,** 450–464 (1965).
6. Sommerfeld, M. & Friedrich, B. Toward Green Ferroalloys: Replacement of Fossil Reductants in the Pre-reduction Process of Chromite by Bio-Based Alternatives. In *REWAS 2022: Developing Tomorrow's Technical Cycles (Volume I),* edited by A. Lazou*, et al.* 1st ed. (Springer, Cham, Switzerland, 2022), pp. 607–619.
7. Wang, Z. *et al.* Thermochemical Behavior of Three Sulfates ($CaSO_4$, K_2SO_4 and Na_2SO_4) Blended with Cement Raw Materials (CaO-SiO_2-Al_2O_3-Fe_2O_3) at High Temperature. *J Anal Appl Pyrolysis* **142,** 104617; 10.1016/j.jaap.2019.05.006 (2019).
8. Yao, X. *et al.* Experimental Investigation of Physicochemical and Slagging Characteristics of Inorganic Constituents in Ash Residues from Gasification of Different Herbaceous Biomass. *Energy* **198,** 117367; 10.1016/j.energy.2020.117367 (2020).
9. Nowakowski, D. J., Jones, J., Brydson, R. & Ross, A. Potassium Catalysis in the Pyrolysis Behaviour of Short Rotation Willow Coppice. *Fuel* **86,** 2389–2402; 10.1016/j.fuel.2007.01.026 (2007).
10. Spokas, K. A. *et al.* Qualitative Analysis of Volatile Organic Compounds on Biochar. *Chemosphere* **85,** 869–882; 10.1016/j.chemosphere.2011.06.108 (2011).
